# Enhancement of co-production of lutein and protein in *Chlorella sorokiniana* FZU60 using different bioprocess operation strategies

**DOI:** 10.1186/s40643-021-00436-9

**Published:** 2021-08-30

**Authors:** Ruijuan Ma, Zhen Zhang, Zhuzhen Tang, Shih-Hsin Ho, Xinguo Shi, Lemian Liu, Youping Xie, Jianfeng Chen

**Affiliations:** 1 Technical Innovation Service Platform for High Value and High Quality Utilization of Marine Organism, Fuzhou University, Fuzhou, 350108 China; 2 Fujian Engineering and Technology Research Center for Comprehensive Utilization of Marine Products Waste, Fuzhou University, Fuzhou, 350108 China; 3 Fuzhou Industrial Technology Innovation Center for High Value Utilization of Marine Products, Fuzhou University, Fuzhou, 350108 China; 4 State Key Laboratory of Urban Water Resource and Environment, School of Environment, Harbin Institute of Technology, Harbin, 150090 China

**Keywords:** *Chlorella sorokiniana*, Co-production, Lutein, Protein, Strategy

## Abstract

Co-production of multiple compounds is an efficient approach to enhance the economic feasibility of microalgae-based metabolites production. In this study, *Chlorella sorokiniana* FZU60 was cultivated under different bioprocess strategies to enhance the co-production of lutein and protein. Results showed that both lutein and protein content (7.72 and 538.06 mg/g, respectively) were highest at the onset of nitrogen deficiency under batch cultivation. Semi-batch III strategy, with 75% microalgal culture replacement by fresh medium, obtained similar content, productivity, and yield of lutein and protein as batch cultivation, demonstrating that it can be used for stable and continuous production. Fed-batch II strategy, feeding with 1/3 modified BG11 medium, achieved super-high lutein and protein yield (28.81 and 1592.77 mg/L, respectively), thus can be used for high-output production. Besides, two-stage strategy, combining light intensity shift and semi-batch cultivation, gained extremely high lutein and protein productivity (15.31 and 1080.41 mg/L/day, respectively), thereby is a good option for high-efficiency production. Moreover, the fed-batch II and two-stage strategy achieved high-quality lutein and protein, thus are promising for the co-production of lutein and protein in *C. sorokiniana* FZU60 for commercial application.

**Supplementary Information:**

The online version contains supplementary material available at 10.1186/s40643-021-00436-9.

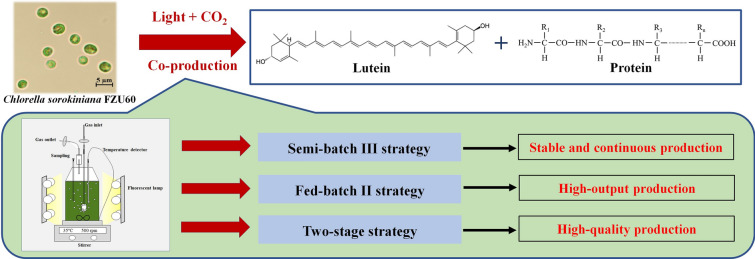

## Introduction

Microalgae are deemed as potential feedstocks of many valuable products, such as proteins, pigments, lipids, and carbohydrates (Dixon and Wilken 2018). However, traditional microalgae cultivation is limited to a single product, leading to low production efficiency and high production cost. In recent years, co-production of multiple compounds from microalgae is regarded as an efficient way to enhance the economic feasibility (Ma et al. 2020a). To date, some microalgae have been used to co-produce multiple compounds, such as co-production of eicosapentaenoic acid (EPA) and fucoxanthin in *Chaetoceros gracilis* (Tachihana et al. 2020), fucoxanthin and stearidonic acid (SDA) in *Isochrysis zhangjiangensis* (Li et al. 2019), protein and carotenoids in *Dunaliella salina* (Sui et al. 2019), and fatty acid and lutein in *Chlamydomonas* sp. JSC4 (Ma et al. 2020b).

Lutein is a carotenoid with human health benefits of preventing cancers, age-related macular degeneration (AMD), and cardiovascular diseases (Lin et al. 2015). It was reported that the global lutein market size was USD 288.41 million in 2019, and is expected to USD 463.16 million by 2027 at a CAGR of 6.10% (Maximize Market Research PVT. LTD. 2020). Microalgae are considered as a promising alternative to the traditional lutein source (marigold) with the advantages of fast-growing, capacity of being industrially scaled up, and less susceptible to climatic, seasonal, and land limitations (Xie et al. 2021). However, microalgae-derived lutein has not been in commercial production due to the high production cost, which is mainly attributed to the relatively low lutein content (normally lower than 1% DW) in microalgal biomass (Sun et al. 2016). Hence, co-production of other compounds could be an efficient way to add value to the lutein production, thus reducing the production cost.

Protein is one of the main components in microalgal cells, accounting for 6% to 70% of dry biomass (50% for most microalgae) in dependence on the species and cultivation conditions (Levasseur et al. 2020). Moreover, the amino acid profile of microalgae perfectly matches the human requirements in FAO/WHO reference (Becker 2007; Kent et al. 2015). Thus, microalgal biomass arises as a potential source of high-quality non-animal protein (Geada et al. 2021). On the other hand, like lutein, protein is a primary metabolite, thereby can accumulate simultaneously with lutein (Ma et al. 2020a). Besides, protein and lutein are hydrosoluble and liposoluble molecules, respectively. These two types of molecules are easily separated, which is beneficial for extraction (Lu et al. 2019; Soto-Sierra et al. 2020). Herein, protein can be used as a superior coproduct during microalgae-derived lutein production for improving economic feasibility. However, few attempts have been made to co-produce lutein and protein in microalgae.

In microalgae-based metabolites production, batch cultivation is the simplest culture mode. However, adverse conditions, such as nutrient and light limitation, occur with the extension of culture time, retarding cell growth and metabolites accumulation. For avoidance of these adverse circumstances, various cultivation strategies have been explored to improve metabolites production in microalgae. For example, semi-batch strategy, refreshing microalgal culture termly, can refrain microalgae from light limitation and sustain log phase growth with stable and continuous high productivity (Ho et al. 2014). Fed-batch strategy, providing nutrient to microalgal culture regularly, can avoid nutrient limitation in microalgal culture, thus obtaining high biomass concentration (Xie et al. 2013). In addition, two-stage strategy is normally performed to obtain enhanced cell growth in first stage and increased metabolite accumulation in second stage for achieving high production and productivity (Zhao et al. 2021). These bioprocess operation strategies would be efficient for the co-production of multiple products in microalgae. For instance, a two-stage strategy was used to enhance the co-production of protein and β-carotene in *Dunaliella salina* (Sui et al. 2019). Likewise, a semi-batch strategy was developed for increased co-production of fucoxanthin and docosahexaenoic acid (DHA) (Sun et al. 2019).

The green microalga *Chlorella* has been widely used for production of lutein (Khoo et al. 2021) or protein (Bin Azmi et al. 2021; Koyande et al. 2020) in recent years. However, the potential of co-production these two compounds from *Chlorella* needs to be evaluated. In this study, a lutein-enriched microalga *Chlorella sorokiniana* FZU60 (Xie et al. 2019a) was cultivated under the phototrophic conditions to investigate its potential for the co-production of lutein and protein. Further, different cultivation strategies, including semi-batch, fed-batch, and two-stage strategy, were carried out to enhance the co-production. The aim is to provide valuable information for improving the economic feasibility of lutein production by co-production of value-added byproducts.

## Materials and methods

### Microalgal strain

*Chlorella sorokiniana* FZU60, isolated from the coastal area of Fujian Province, China, was maintained in modified BG11 medium algal plate (deposited at 25 °C). For pre-culture, microalga was inoculated from the algal plate, and cultivated in 1-L photobioreactor with 1-L modified BG11 medium for 3 days. The seed culture was cultivated at 30 °C under the continuous fluorescent light of 250 μmol/m^2^/s, agitated at 500 rpm, and aerated with 2.5% CO_2_ at a rate of 0.15 vvm. The recipe of modified BG11 medium was as follows (g/L): NaNO_3_ 0.750, K_2_HPO_4_ 0.030, MgSO_4_·7H_2_O 0.075, citric acid monohydrate 0.006, Na_2_CO_3_ 0.020, CaCl_2_·2H_2_O 0.036, ferric ammonium citrate 0.006, EDTA·2Na 0.001, trace elements 1 mL. The composition of trace elements was as follows (g/L): H_3_BO_3_ 2.860, MnCl_2_·4H_2_O 1.810, ZnSO_4_·7H_2_O 0.222, Na_2_MoO_4_·2H_2_O 0.390, CuSO_4_·5H_2_O 0.079, Co(NO_3_)_2_·6H_2_O 0.049.

### Experimental operation

#### Batch cultivation

The pre-cultured microalga was inoculated into fresh modified BG11 medium at an initial density of 65 mg/L and an initial nitrate concentration of 0.75 g/L. The cultures were cultivated at 35 °C under the continuous fluorescent light of 600 μmol/m^2^/s, agitated at 500 rpm, and aerated with 2.5% CO_2_ at a rate of 0.15 vvm. The microalgal culture was sampled every 0.5 day for the determination of biomass concentration, nitrogen concentration, lutein content, and protein content. The amino acid composition was measured when nitrogen was replete (0.5 days), 90% consumed (1.56 days), and completely depleted (2.5 days).

#### Semi-batch cultivation strategy

*C. sorokiniana* FZU60 was cultivated at the same conditions as batch cultivation until biomass concentration reached around 1.42 g/L. Then, 25%, 50% and 75% algal culture were replaced by the same volume of 1 × modified BG11 medium for semi-batch I, II, and III strategy, respectively. The cultivation proceeded at the same conditions as before the replacement until 90% nitrogen was consumed again, and then a new round of replacement was conducted. The replacement was performed for five rounds. The microalgal culture was sampled at set time intervals for the measurement of biomass concentration, nitrogen concentration, lutein content, and protein content.

#### Fed-batch cultivation strategy

Two types of fed-batch cultivation strategies were carried out to further enhance lutein production. The cultivation conditions were the same as batch cultivation except as specifically described. For fed-batch I and II, concentrated nutrients were fed into microalgal culture to adjust the feeding concentration to be 0.25 g/L sodium nitrate and 1/3 modified BG11 medium, respectively, at the onset of nitrogen depletion (90% nitrogen was consumed). The cultivation lasted for 5.5 and 7.5 days for fed-batch I and II, respectively. Microalgal culture was sampled at set time intervals for the measurement of biomass concentration, nitrogen concentration, lutein content, and protein content.

#### Two-stage cultivation strategy

For the two-stage cultivation strategy, *C. sorokiniana* FZU60 was initially cultivated at the same conditions as batch cultivation except that the light intensity was set at 800 μmol/m^2^/s, which was denoted as stage I. At the beginning of nitrogen depletion, 5% microalgal culture was inoculated into a 95% fresh modified BG11 medium for the next round of stage I. The remaining microalgal culture was fed with 1 × modified BG11 medium and then cultivated under 250 μmol/m^2^/s fluorescent light until nitrogen depletion, which was denoted as stage II. This two-stage cultivation was repeated five times. The microalgal culture was sampled at an interval of 0.5 day for the determination of biomass and nitrogen concentration. Lutein and protein content were measured whenever nitrogen was at the onset of depletion.

### Analysis methods

#### Determination of growth and nitrate concentration

A spectrophotometer (U-2001, Hitachi, Tokyo, Japan) was used to measure the optical density of 682 nm (OD_682_) of microalgal culture. Biomass concentration was determined by Eq. (1):

1 $$y = 0.2244 x - 0.0045 (R^{2} = 0.9970),$$

where *y* and *x* are biomass concentration and OD_682_, respectively.

The biomass productivity (*P*_biomass_) was determined by Eq. (2):

2 $$P_{{{\text{biomass}}}} \left( {{\text{g}}/{\text{L}}/{\text{day}}} \right) = \frac{{X_{t} - X_{0} }}{t},$$

where *X*_*t*_ is the biomass concentration (g/L) on t days, *X*_*0*_ is the initial biomass concentration (g/L), and *t* is culture time (day).

Nitrate concentration was measured as previously reported (Xie et al. 2019b). Briefly, 10 mL microalgal culture was filtered through a cellulose acetate filter with 0.22 μm pore size. After properly diluted, the supernatant was measured at the optical density of 220 nm (OD_220_). The calculation of nitrate concentration was conducted by Eq. (3):

3 $$y = 22.63 x + 0.0359(R^{2} = 0.9990),$$

where *y* is nitrate concentration, and *x* is OD_220_.

#### Determination of lutein accumulation

Extraction and quantification of carotenoids were performed as previously reported (Xie et al. 2019b). The lutein productivity (*P*_lutein_) and lutein yield (*Y*_lutein_) were determined by Eq. (4) and Eq. (5), respectively:

4 $$P_{{{\text{lutein}}}} ({\text{mg}}/{\text{L}}/{\text{day}}) = C_{{{\text{lutein}}}} \times P_{{{\text{biomass}}}} ,$$

5 $$Y_{{{\text{lutein}}}} ({\text{mg}}/{\text{L}}) = C_{{{\text{lutein}}}} \times C_{{{\text{biomass}}}} ,$$

where *P*_biomass_ (g/L/day), *C*_biomass_ (g/L), and *C*_lutein_ (mg/g) are the biomass productivity, biomass concentration, and lutein content, respectively.

#### Determination of protein accumulation

Protein extraction was performed by using a protein extraction kit (BB-3131-1, BestBio, Shanghai, China). The measurement of protein content was performed by a Pierce® BCA protein assay kit (Thermo Scientific, Waltham, MA, USA). The protein productivity (*P*_protein_) and protein yield (*Y*_protein_) were measured by Eq. (6) and Eq. (7), respectively:

6 $$P_{{{\text{protein}}}} ({\text{mg}}/{\text{L}}/{\text{day}}) = C_{{{\text{protein}}}} \times P_{{{\text{biomass}}}} ,$$

7 $$Y_{{{\text{protein}}}} ({\text{mg}}/{\text{L}}) = C_{{{\text{protein}}}} \times C_{{{\text{biomass}}}} ,$$

where *P*_biomass_ (g/L/day), *C*_biomass_ (g/L), and *C*_protein_ (mg/g) are the biomass productivity, biomass concentration, and protein content, respectively.

The amino acid composition was determined as previously described (Tong et al. 2009) with modification. In short, 25 mg freeze-dried microalga was mixed thoroughly with 10 mL 6 M HCl and three drops of phenol under nitrogen gas, and then hydrolyzed at 110 °C for 22 h. The hydrolysate was cooled down to room temperature and filtered through a 0.22-μm nylon filter. Subsequently, 1 mL of the hydrolysate was dried at 50 °C using a vacuum desiccator, dissolved in 1 mL water, and then dried again. Hydrolyzed protein was dissolved in 1 mL sodium citrate buffer (pH 2.2), and subsequently filtered through a 0.22-μm nylon filter, which was used for the determination of amino acid content. An automatic amino acid analyzer (Biochrom 30 + series, Biochrom, Cambridge, UK) was used to identify and quantify amino acids.

The essential amino acid index (EAAI) was determined as previously described (Sui et al. 2019) by Eq. (8):

8 $${\text{EAAI}} = \sqrt[n]{{\frac{{{\text{aa}}1}}{{{\text{AA}}1}} \times \frac{{{\text{aa}}1}}{{{\text{AA}}1}} \times \cdots \times \frac{{{\text{aa}}n}}{{{\text{AA}}n}}}}$$

where aa*n* and AA*n* are the proportion of a certain essential amino acid content in total protein content (mg/g) in the sample and FAO/WHO reference (Joint WHO/FAO/UNU Expert Consultation 2007), respectively.

### Statistical analysis

The results in tables and figures are presented as the means ± standard deviation (SD) from three replicates. Origin v9.0 software (OriginLab Inc., Northampton, Mass, USA) was used to perform statistical analyses, and *p* < 0.05 were considered statistically significant.

## Results and discussion

### Co-production of lutein and protein in* C. sorokiniana *FZU60 under batch cultivation

**Fig. 1:**
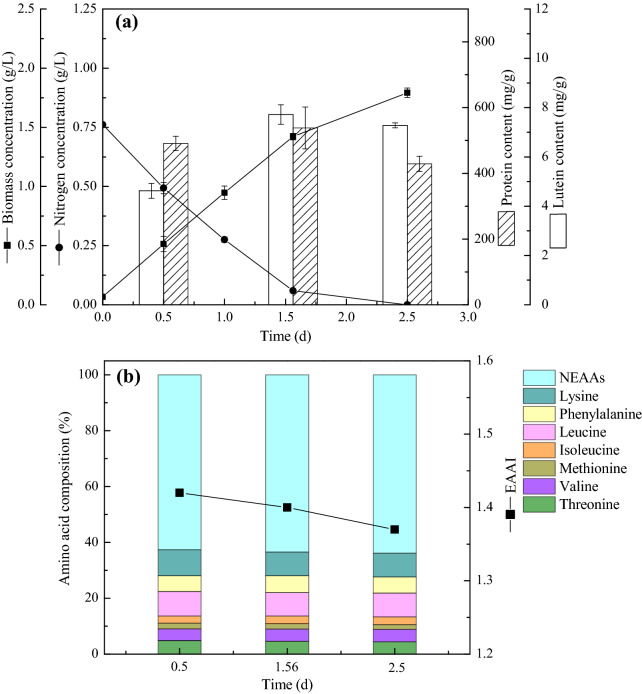
Cell growth and lutein and protein accumulation of *C. sorokiniana* FZU60 under batch cultivation. **a** Biomass concentration, nitrogen concentration, lutein content, and protein content; **b** amino acid composition

As shown in Fig. 1a, for batch cultivation, lutein and protein content increased with the increase in biomass when nitrogen was replete (day 0.5 to 1.56), while decreased after nitrogen was depleted (day 2.5). It was reported that xanthophyll and protein could only be accumulated in microalgae under the condition of sufficient nitrogen (Chen et al. 2015; Xie et al. 2013). This could be due to that algal cells absorb sufficient nitrogen for the biosynthesis of nitrogen-containing metabolites, such as protein. In addition, as a primary carotenoid, lutein is growth-related due to its function in light-harvesting and nonphotochemical quenching (NPQ) as a structural pigment bound to light-harvesting complex (LHC) associated proteins (Xie et al. 2021). Since nitrogen was a critical factor for cell growth (Lee et al. 2015; Plyusnina et al. 2020; Vello et al. 2018), both lutein and protein content increased concomitantly with cell growth under sufficient nitrogen. On the other hand, cells prefer to synthesize high-energy compounds, such as starch and lipid, under nitrogen deficiency conditions for energy store to overcome adverse environment (Siaut 2011). Thus, primary metabolites including protein and lutein may be degraded and shifted to those high-energy compounds when nitrogen was depleted.

To be noted, lutein and protein content reached 7.72 and 538.06 mg/g on day 1.563, respectively. The newly isolated microalga *C. sorokiniana* FZU60 was deemed as a good candidate for lutein production (Xie et al. 2019a). Nevertheless, the production cost is considerably high attributed to the relatively low lutein content (normally lower than 1%) in cell composition. Comprehensive utilization of algal biomass for multiple metabolites production can be used as an efficient way to improve the economic feasibility of lutein production (Ma et al. 2020a). This study found that the proportion of protein in cell composition was higher than 50%, indicating that *C. sorokiniana* FZU60 is a good candidate for the co-production of lutein and protein.

The high protein content in *C. sorokiniana* FZU60 demonstrated that it could be used as a protein source for food or feed industry. Thus, it is essential to investigate the amino acid composition of *C. sorokiniana* FZU60, which is an important indicator for protein quality. As shown in Fig. 1b, the proportion of EAAs decreased from 37.37% to 36.18% with the consumption of nitrogen. EAAs cannot be biosynthesized by humans and have to be provided from food, thus EAAs proportion is a crucial marker for nutritional value (Muys et al. 2019). The EAAs proportion of *C. sorokiniana* FZU60 is in a relatively high level compared with some microalgae, such as *Spirulina* sp. (Prates et al. 2020). Concomitantly, EAAI dropped from 1.42 to 1.37 from day 0.5 to 2.5. Protein with EAAI higher than one is considered as a good-quality nutrient for human consumption (Kent et al. 2015; Sui et al. 2019). The EAAI of *C. sorokiniana* FZU60 was superior to many microalgae, such as *Nannochloropsis granulata* (Tibbetts et al. 2015), *Dunaliella salina* (Sui et al. 2019), and many *Chlorella* and *Spirulina* species (Muys et al. 2019). In this study, lutein and protein content were highest at the onset of nitrogen depletion when EAAI was 1.40, indicating that protein can be a high-quantity and high-quality coproduct from lutein production in *C. sorokiniana* FZU60.

### Co-production of lutein and protein in *C. sorokiniana* FZU60 under semi-batch strategy

**Fig. 2:**
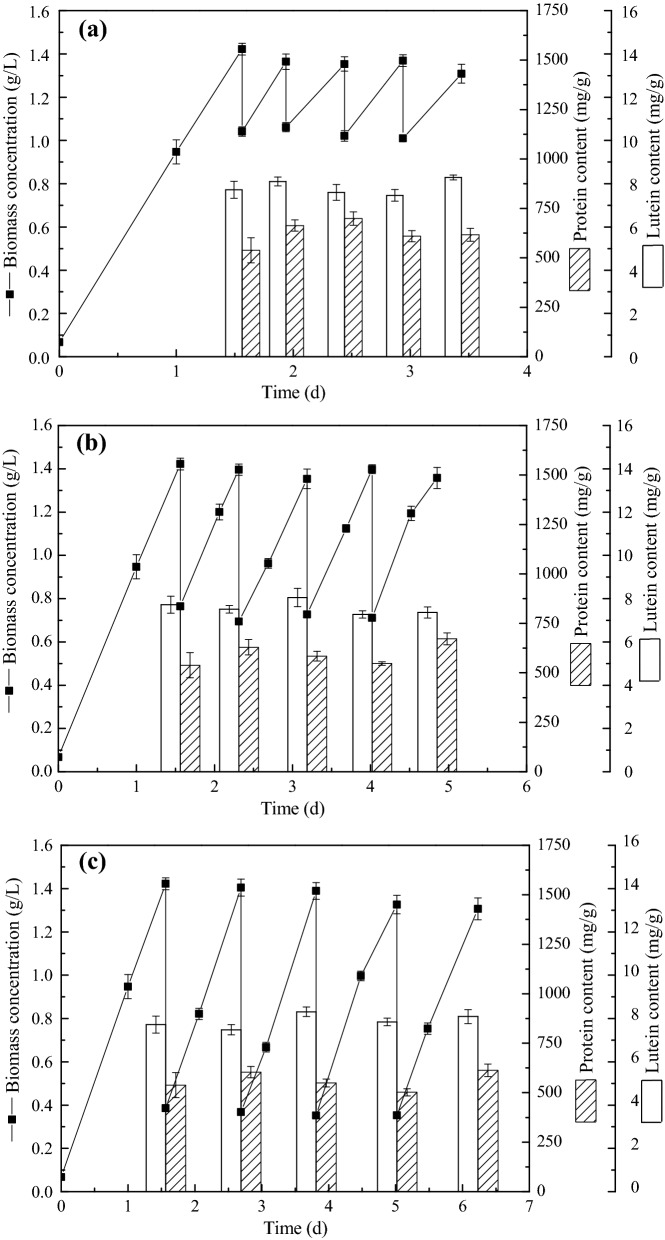
Cell growth and lutein and protein accumulation of *C. sorokiniana* FZU60 under semi-batch cultivation strategies. **a** Semi-batch I strategy; **b** semi-batch II strategy;** c **semi-batch III strategy

In batch cultivation, with the increase in biomass concentration and the consumption of nutrients, the growth of algal cells would be retarded by the limitation of light and nutrients, and this problem could be effectively solved by semi-batch cultivation (Ferreira et al. 2011). Semi-batch cultivation can achieve stable and continuous production, and avoid restarting microalgae cultivation constantly, which can bring down contamination problem and production cost (Fuentes-Grünewald et al. 2015). In this study, three types of semi-batch cultivation strategies, with 25%, 50%, and 75% microalgal culture replacement by fresh medium, respectively, were carried out to improve the co-production of lutein and protein in *C. sorokiniana* FZU60. As shown in Fig. 2, biomass concentration, lutein content, and protein content were relatively stable in each round, indicating that stable and continuous production was achieved by semi-batch cultivation. In addition, biomass, lutein, and protein productivity (852.50, 6.75, and 478.70 mg/L/day, respectively) of semi-batch III were relatively superior to semi-batch I and II (Table 1). Similar results were found in *C. sorokiniana* MB-1-M12 that higher biomass and lutein productivity were obtained under 75% replacement in semi-batch cultivation, compared with that of 25% and 50% replacement (Chen et al. 2019). This could be due to that an increase in initial inoculum size (low replacement) leads to a reduction in cell growth rate (Lu et al. 2012). Moreover, the content, productivity, and yield of lutein and protein in semi-batch III were close to that of batch cultivation, indicating that this semi-batch strategy can be used to co-produce lutein and protein stably and continuously.

**Table 1:** Co-production of lutein and protein in *C. sorokiniana* FZU60 under different cultivation strategies

Cultivation strategy	Biomass productivity (mg/L/day)	Lutein content (mg/g)	Lutein productivity (mg/L/day)	Lutein yield (mg/L)	Protein content (mg/g)	Protein productivity (mg/L/day)	Protein yield (mg/L)
Batch	852.76 ± 44.55	7.72 ± 0.40	6.58 ± 0.45	10.99 ± 0.77	538.06 ± 63.60	458.83 ± 23.97	766.96 ± 32.34
Semi-batch I	718.09 ± 134.24	7.83 ± 0.35	5.67 ± 1.09	10.67 ± 0.40	625.47 ± 60.98	446.18 ± 77.46	851.55 ± 30.42
Semi-batch II	805.46 ± 42.58	7.58 ± 0.31	6.11 ± 0.35	10.50 ± 0.43	594.14 ± 56.51	478.14 ± 44.24	822.30 ± 23.28
Semi-batch III	852.50 ± 55.06	7.89 ± 0.32	6.75 ± 0.51	10.80 ± 0.47	561.27 ± 46.67	478.70 ± 52.55	768.59 ± 20.76
Fed-batch I	439.52 ± 17.55	8.96 ± 0.26	3.94 ± 0.27	15.57 ± 2.90	438.31 ± 43.96	192.26 ± 11.63	881.49 ± 35.62
Fed-batch II	611.20 ± 19.94	10.51 ± 0.26	6.43 ± 0.37	28.81 ± 1.50	479.59 ± 9.14	293.03 ± 3.98	1592.77 ± 56.93
Two-stage	1710.71 ± 97.13	8.74 ± 0.40	15.31 ± 1.27	22.93 ± 1.87	632.00 ± 42.84	1080.41 ± 87.11	1617.79 ± 77.35

### Co-production of lutein and protein in *C. sorokiniana* FZU60 under fed-batch strategy

**Fig. 3:**
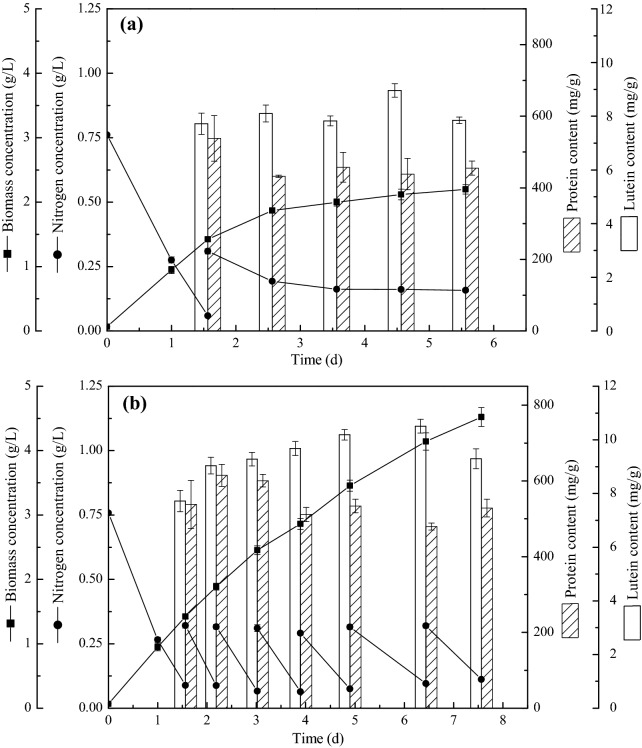
Cell growth and lutein and protein accumulation of *C. sorokiniana* FZU60 under fed-batch cultivation strategies. **a** Fed-batch I strategy; **b** fed-batch II strategy

Fed-batch cultivation is an easily operated strategy to avoid nutrient deficiency during microalgal cultivation. Nitrogen was considered as a crucial nutrient for lutein accumulation in microalgae, and lutein content significantly increased when feeding with relatively low concentration of nitrogen (Xie et al. 2013). Besides, as a nitrogen-containing compound, protein accumulation highly depends upon nitrogen. However, whether the other nutrients are required for lutein and protein accumulation during fed-batch cultivation needs to be further investigated. In view of this, two types of fed-batch strategies feeding with 0.25 g/L sodium nitrate and 1/3 modified BG11 medium, respectively, at the beginning of nitrogen deficiency, were carried out in this study. As shown in Fig. 3a, nitrogen consumption rate significantly slowed down after feeding with 0.25 g/L sodium nitrate in fed-batch I. The nitrogen concentration was almost plateaued and kept at 0.16 g/L after day 3.56, indicating that it was not consumed anymore. However, nitrogen was regularly consumed in fed-batch II, and six times of 1/3 modified BG11 medium feeding were accomplished on day 7.56 (Fig. 3b). This could be due to that the lack of other nutrients reduced nitrogen consumption in microalgal cells (Osorio et al. 2019). It was reported that some algae could only maintain a normal nitrogen consumption rate under sufficient nutrient conditions, such as inorganic salts and trace elements (Crawford et al. 2003). Consistently, biomass concentration slowly increased and then levelled off in fed-batch I, while continually raised during all the cultivation period in fed-batch II. Medium with all components instead of sole nutrient was generally used as feeding nutrients in fed-batch strategy for improving cell growth and metabolites accumulation in microalgae, such as *Chlamydomonas reinhardtii* (Fields et al. 2018), *Scenedesmus incrassatulus* (Garcia-Canedo et al. 2016), and *Phaeodactylum tricornutum* (Chauton et al. 2013). This can avoid nutrient limitation since all components of the medium are consumed during the cultivation.

On the other hand, lutein content showed an upward trend with the time course of cultivation, followed by a decrease at the end. In contrast, protein content was highest at the beginning of cultivation, decreased and then levelled off afterwards. The increase in lutein content during the cultivation could be due to that the light intensity per algal cell decreased with the increase in biomass concentration. Thus, algal cells synthesized more lutein to capture light energy (Dineshkumar et al. 2016; Vaquero et al. 2014). Additionally, protein is a primary metabolite, which is growth related. Hence, protein content was at a high level at the primary stage of cultivation when cells grew extremely fast, and then decreased with the reduction in cell growth rate. To be noted, the maximal lutein content reached 10.51 mg/g with a high biomass concentration of 4.14 g/L in fed-batch II, leading to super-high lutein and protein yield of 28.81 and 1592.77 mg/L, respectively (Table 1). These results were significantly greater than that of batch cultivation, semi-batch strategy, and fed-batch strategy.

### Co-production of lutein and protein in *C. sorokiniana* FZU60 under two-stage strategy

In some cases, the trends of cell growth and metabolites accumulation are contradictory in microalgae. Thus, a two-stage strategy is normally explored to obtain high biomass and metabolite content separately. For example, in *Haematococcus pluvialis*, high cell density was initially achieved under sufficient nutrient and low light conditions, and then cells were exposed to stressed conditions, such as nitrogen starvation, high light, and salinity stress, to induce astaxanthin accumulation (Zhao et al. 2019). Similarly, a hetero-photoautotrophic two-stage strategy was used to obtain high biomass concentration and subsequently fucoxanthin accumulation in *Nitzschia laevis* (Lu et al. 2018). For most lutein-producing microalgae, cell growth rate increases while lutein content reduces with an increase in light intensity (Ma et al. 2018; Xie et al. 2013), which is unbeneficial for lutein production. Similar result was found in the previous study that biomass productivity increased, while lutein content decreased, with the increase in light intensity from 250 to 800 μmol/m^2^/s, resulting in that peaked lutein productivity was obtained at 600 μmol/m^2^/s in *C. sorokiniana* FZU60 (Additional file 1: Figure S1). Thus, a two-stage strategy combining light intensity shift and semi-batch cultivation was explored to enhance lutein production in *C. sorokinia*na FZU60.

**Fig. 4:**
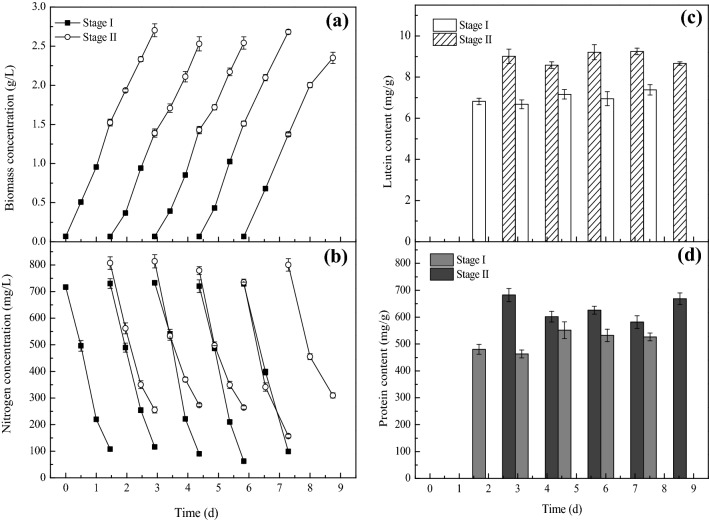
Cell growth and lutein and protein accumulation of *C. sorokiniana* FZU60 under two-stage cultivation strategy. **a** Biomass concentration; **b** nitrogen concentration; **c** lutein content; **d **protein content

As shown in Fig. 4, biomass concentration, nitrogen consumption, lutein content, and protein content were at a relatively stable level for each round of cultivation. Noticeably, lutein and protein content significantly increased in stage II compared with that of stage I. This could be due to that the sudden drop in light intensity led to the synthesis of more LHC-associated proteins for the capture of light energy in microalgal cells. A similar result was found in *Scenedesmus obliquus* FSP-3 that lutein content was significantly enhanced when light intensity shift from 300 μmol/m^2^/s to 75 μmol/m^2^/s (Ho et al. 2015). To be noted, high protein content (632 mg/g) was obtained in the two-stage strategy with a high level of lutein content (8.74 mg/g). Moreover, extremely high biomass productivity (1710.71 mg/L/day) was obtained in the two-stage strategy, leading to super-high lutein and protein productivity (15.31 and 1080.41 mg/L/day, respectively) (Table 1). Besides, compared with fed-batch II strategy, the shift of light intensity from high to low together with the shorter cultivation period indicated that the two-stage strategy was more energy-efficient. Herein, the two-stage strategy is high-efficiency for the co-production of lutein and protein.

### Comparison of different cultivation strategies

Based on the above results, different cultivation strategies have their pros and cons. Semi-batch III strategy obtained similar content, productivity, and yield of lutein and protein as batch cultivation, which can be used for stable and continuous co-production of lutein and protein. Fed-batch II, an easily operated strategy, achieved high lutein and protein yield, thus can be used for high-output co-production of lutein and protein. Besides, extremely high lutein and protein productivity were gained in the energy-efficient two-stage strategy with relatively high levels of lutein and protein yield. Hence, the two-stage strategy is a good option for high-efficiency co-production of lutein and protein.

**Fig. 5:**
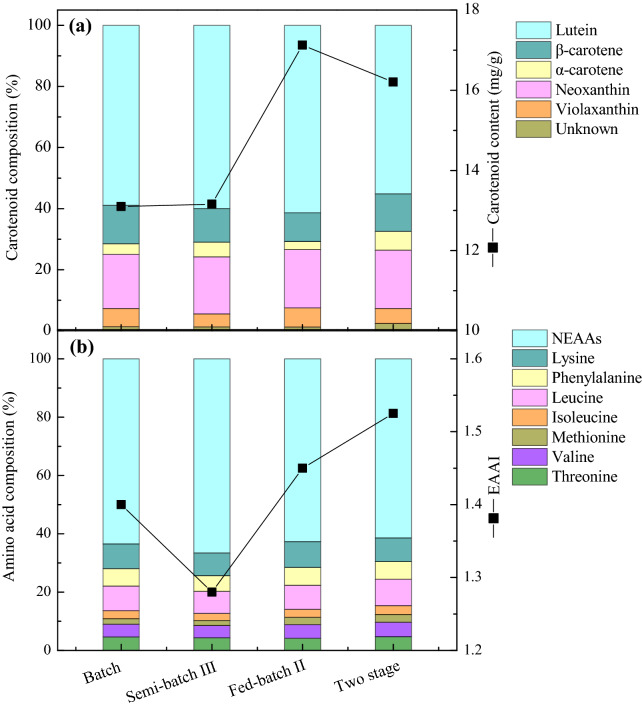
Carotenoid and amino acid composition in *C. sorokiniana* FZU60 under different cultivation strategies.** a **Carotenoid composition;** b** amino acid composition

In addition, carotenoid and amino acid composition need to be investigated, since they are important for the quality of lutein and protein. As shown in Fig. 5, carotenoid content was highest in fed-batch II strategy, followed by two-stage strategy, and it was lowest in batch cultivation. Lutein accounted for about 60% of the total carotenoids under different cultivation modes, and fed-batch II strategy obtained the highest lutein proportion (61.38%). The highest EAAs proportion (38.61%) was obtained in two-stage strategy, followed by fed-batch II strategy, and it was lowest in semi-batch III strategy. Consistently, the EAAI value was in the order of two-stage > fed-batch II > batch > semi-batch III. In conclusion, high-quality lutein and protein were obtained in fed-batch II and two-stage strategy. Moreover, high yield or productivity of lutein and protein were achieved by these two strategies, respectively. Herein, fed-batch II and two-stage strategy are superior for the co-production of high-quality lutein and protein.

## Conclusions

For batch cultivation, the highest lutein and protein content were obtained at the onset of nitrogen deficiency. Semi-batch III strategy can be used for stable and continuous co-production of lutein and protein. Fed-batch II strategy is advantageous for high-output co-production of lutein and protein with super-high yield. Additionally, two-stage strategy is a good option for high-efficiency co-production of lutein and protein with extremely high productivity. Moreover, high-quality lutein and protein were obtained under fed-batch II and two-stage strategy, indicating that these two strategies are superior for the co-production of lutein and protein in *C*. *sorokiniana* FZU60. This study provides new insight into the improvement of economic feasibility of lutein production by co-producing high-value protein.

### Supplementary Information

**Additional file 1: Figure S1.** Effect of light intensity on cell growth and lutein accumulation. (a) Biomass productivity and maximum specific growth rate; (b) lutein content and productivity. **Figure S2.** Effect of temperature on cell growth and lutein accumulation. (a) Biomass productivity and maximum specific growth rate; (b) lutein content and productivity.

## Data Availability

The datasets used and/or analyzed during the current study are available from the corresponding author on reasonable request.

## Abbreviations

AMD: Age-related macular degeneration
DHA: Docosahexaenoic acid
EAAs: Essential amino acids
EAAI: Essential amino acid index
EPA: Eicosapentaenoic acid
LHC: Light-harvesting complex
NEEAs: Nonessential amino acids
NPQ: Nonphotochemical quenching
SD: Standard deviation
SDA: Stearidonic acid
