# Supplementary material for: Enhancement of co-production of lutein and protein in Chlorella sorokiniana FZU60 using different bioprocess operation strategies
Source: Bioresour Bioprocess. 2021 Aug 30;8(1):82. doi: 10.1186/s40643-021-00436-9 (PMC10992755; doi:10.1186/s40643-021-00436-9)
Supplement: Supplementary file 1 — Additional file 1: Figure S1. Effect of light intensity on cell growth and lutein accumulation. (a) Biomass productivity and maximum specific growth rate; (b) lutein content and productivity. Figure S2. Effect of temperature on cell growth and lutein accumulation. (a) Biomass productivity and maximum specific growth rate; (b) lutein content and productivity. [file 40643_2021_436_MOESM1_ESM.docx]

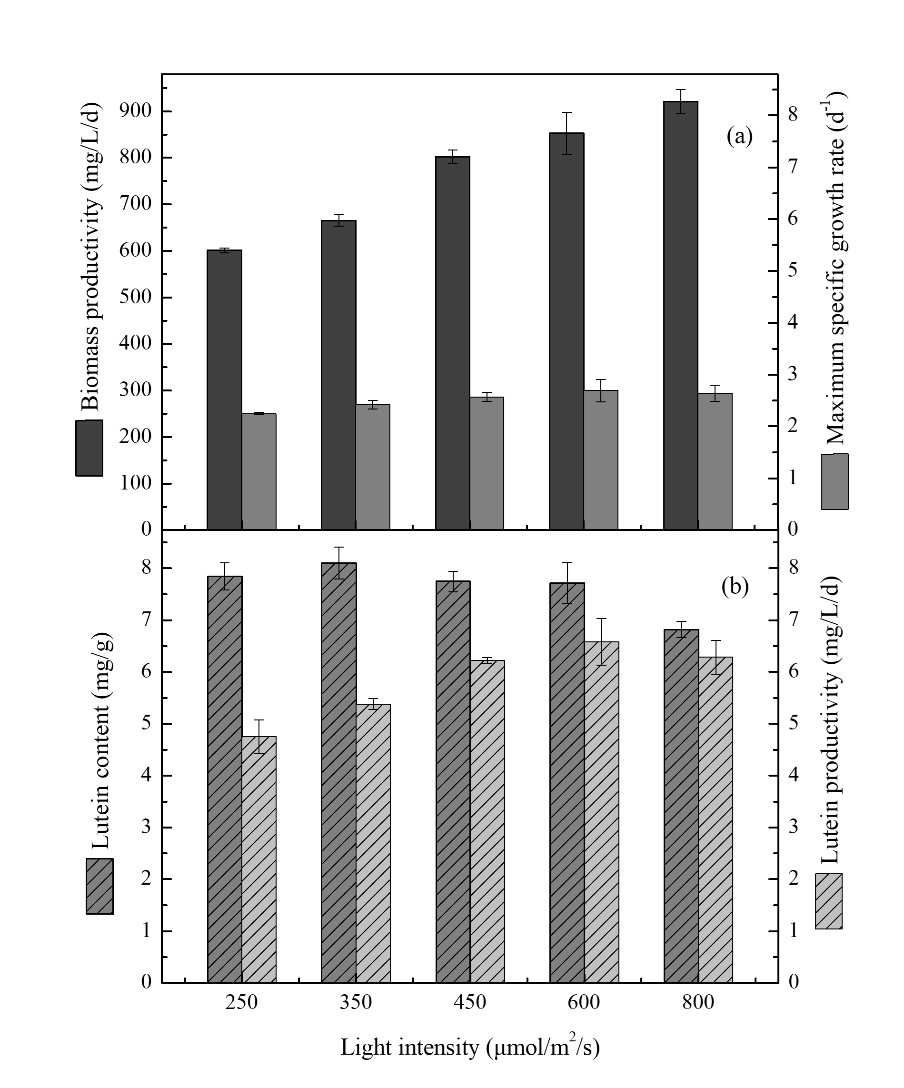


Figure S1 Effect of light intensity on cell growth and lutein accumulation. (a) Biomass productivity and maximum specific growth rate; (b) lutein content and productivity.


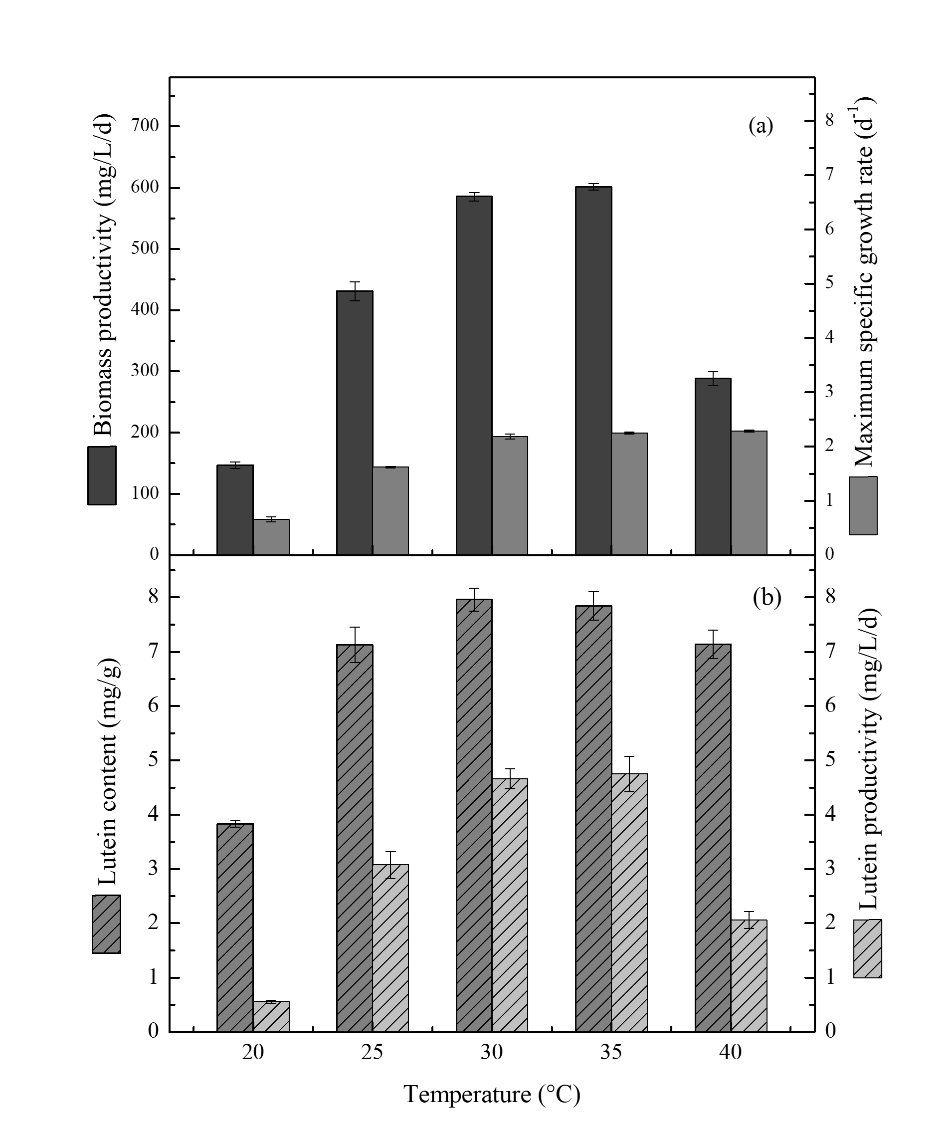


Figure S2 Effect of temperature on cell growth and lutein accumulation. (a) Biomass productivity and maximum specific growth rate; (b) lutein content and productivity.
